# Interleukin-18 Antagonism Improved Histopathological Conditions of Malaria Infection in Mice

**Published:** 2015

**Authors:** Marzieh JABBARZARE, Voon Kin CHIN, Herni TALIB, Mun Fei YAM, Siti Khadijah ADAM, Haniza HASSAN, Roslaini ABDUL MAJID, Che Norma MAT TAIB, Mohamad Aris MOHD MOKLAS, Mohamad TAUFIK HIDAYAT, Hasidah MOHD SIDEK, Rusliza BASIR

**Affiliations:** 1 *Dept. of Human Anatomy, Faculty of Medicine & Health Sciences, Universiti Putra Malaysia, Selangor, Malaysia*; 2 *Dept. of Biomedical Sciences, Faculty of Medicine & Health Sciences, Universiti Putra Malaysia, Selangor, Malaysia*; 3 *Dept. of Pathology, Faculty of Medicine & Health Sciences, Universiti Putra Malaysia, Selangor, Malaysia*; 4 *School of Pharmaceutical Sciences, Universiti Sains Malaysia, Penang, Malaysia*; 5 *Dept. of Medical Microbiology and Parasitology, Faculty of Medicine & Health Sciences, Universiti Putra Malaysia, Selangor, Malaysia*; 6 *School of Biosciences and Biotechnology, Faculty of Science and Technology, Universiti Kebangsaan Malaysia, Malaysia*

**Keywords:** *Malaria*, *Interleukin-18*, *Plasmodium berghei*, *Histopathology*

## Abstract

***Background:*** Interleukin 18 (IL-18) exerts pleiotropic roles in many inflammatory-related diseases including parasitic infection. Previous studies have demonstrated the promising therapeutic potential of modulating IL-18 bioactivity in various pathological conditions. However, its involvement during malaria infection has yet to be established. In this study, we demonstrated the effect of modulating IL-18 on the histopathological conditions of malaria infected mice.

***Methods:*** *Plasmodium berghei* ANKA infection in male ICR mice was used as a model for malaria infection. Modulation of IL-18 release was carried out by treatment of malarial mice with recombinant mouse IL-18 (rmIL-18) and recombinant mouse IL-18 Fc chimera (rmIL-18Fc) intravenously. Histopathological study and analysis were performed on major organs including brain, liver, spleen, lungs and kidney.

***Results:*** Treatment with rmIL-18Fc resulted in significant improvements on the histopathological conditions of the organs in malaria-infected mice.

***Conclusion:*** IL-18 is an important mediator of malaria pathogenesis and targeting IL-18 could prove beneficial in malaria-infected host.

## Introduction

Malaria is a disease of parasitic infection caused by the* Plasmodia* parasite that belongs to the *Plasmodiidae *family in the Apicomplex Phylum. There are four medically important *Plasmodium *species known to cause malaria infection in human, namely *P. falciparum*, *P. *vivax*, P. malaria *and* P. ovale* (1). More recently, *P. knowlesi*, a monkey species of malaria parasite, has been recognized to cause zoonotic malaria in human, especially in the Southeast Asian region (2). *P. berghei* ANKA infection in animal model can mimic most of the important clinical aspects of malaria pathogenesis, including the organ-specific and severe disease syndromes (3, 4). 

Interleukin-18 (IL-18), a recently-cloned novel cytokine, was initially recognized as a potent interferon- (IFN-)-inducing factor due to its ability to induce high levels of IFN- secretion from T cells, B cells, NK cells and activated macrophages during inflammation, endotoxic shock and microbial infection, particularly in the presence of IL-12 (5-8). IFN is considered as an important cytokine that contributes towards the host defense mechanism against many disease conditions (9, 10). It functions as an essential regulator of chronic inflammation in human autoimmune diseases (6, 11). Patients with severe initial stage of malaria were found to have elevated levels of IFN- (12). The level of IL-18 is elevated in response to severe *P. falciparum* malaria, suggesting its important involvement in the disease severity, rather than its protective role reported for murine malaria (13). *Plasmodium *parasite invasion into the blood circulation triggers the host immune system to fight against the infection. 

Apart from circulating in the peripheral blood and infecting the RBCs, the parasites may also sequester in the organs (14). During the acute or severe stages of malaria, pathological changes occur in organs such as the liver, spleen, lungs and kidneys (15). With several studies indicating the promising therapeutic potential of modulating IL-18 bioactivity for the treatment of inflammation-related diseases, the present study was designed to elucidate further the role and involvement of IL-18 during malaria infection.

Here, we investigated the effects of modulating IL-18 release and production on the histopathological changes during malaria infection, with the ultimate aim of evaluating the significance of IL-18 role in malaria pathogenesis.

## Materials and Methods

***Animals and parasites***

Male ICR mice (17-20 g) were used throughout the study. The animals were housed at 27-30 ºC and fed on standard diet and drinking water* ad libitum. *The animals were kept in cages covered with mosquito nets to avoid any possible malaria transmission during the experiment. All the procedures conducted on the animals have been approved by the Animal Care and Use Committee (ACUC) of the Faculty of Medicine and Health Sciences, Universiti Putra Malaysia (ethical approval reference number: UPM/FSPK/PADS/BR-UUH/00479). The malaria parasite used in this study was *Plasmodium berghei* ANKA strain originally obtained from The Institute of Medical Research (IMR), Kuala Lumpur, Malaysia. 

***Induction of malaria and parasitaemia measurement***

Malaria infection was initiated by intraperitoneal inoculation of normal mice with 2 x 10^7^ parasitized red blood cells (PRBC) in a 0.2 ml volume. Controls were given an equivalent volume and dilution of normal RBCs. Parasitaemia monitoring and measurement was conducted by means of thin blood film stained with Leishman’s stain and viewed under light microscopy with oil immersion (1000x magnification). The Leishman-positive cells counted were taken as an index of parasitaemia. Five fields containing 200 cells each were counted and parasitaemia was determined as the percentage of the total red blood cells counted that contained the Leishman-positive bodies. The average results from the five different fields were then taken as the final percentage of parasitaemia.

***Treatment of malaria-infected mice with recombinant IL-18 (rmIL-18) and recombinant IL-18 Fc chimera (rmIL-18 Fc chimera)***

The recombinant IL-18 and recombinant IL-18 Fc chimera used in this study were purchased from R&D system, USA. Mice were divided into four different groups (N=8 in each group). **Group 1: **Control mice receiving sterile PBS (C+PBS); **Group II: **Malarial mice treated with sterile PBS (M+PBS); **Group III: **Malarial mice treated with rmIL-18 (M+rmIL-18); **Group IV:** Malarial mice treated with rmIL-18 Fc chimera (M+rmIL-18 Fc chimera). In the malarial groups, the mice were inoculated with *P. berghei* as described in the previous section. Controls received an equivalent volume and dilution of normal RBCs. Treatments were carried out once daily starting at mid-day of day 1, following inoculation with the parasites in the morning, and continued until day 4 post-infection. All drugs were dissolved in sterile PBS. In groups I and II, the control and malarial mice were injected with 0.2 ml sterile PBS intravenously. In groups III and IV, the mice received 2 g (0.2 ml, i.v) of rmIL-18 or rmIL-18 Fc chimera, respectively. On day 5 post inoculation and treatment, all experimental mice were anesthetized by inhalation of diethyl ether and sacrificed for organ collection, which included the brain, liver, lungs, spleen and kidneys. The organs were immediately placed in formalin after harvested. Day 5 was chosen for organ harvesting to avoid the high mortality rate of infected mice on day 6. The organs were subjected to a tissue processing cycle in an automated tissue processor (Leica, Germany), and then embedded into melted paraffin wax using a histoembedder (Leica, Germany). The embedded tissues were sectioned into 4.0μm-thick slices with a microtome (Leica, Germany), and stained with hematoxylin and eosin (H&E) in an autostainer (Leica, Germany). The morphological alterations in the tissues of the control and treated malarial mice were observed under light microscopy at 100x, 200x and 400x magnifications. 

## Results

For histopathological examination on treatment with IL-18 related drugs, the malaria-infected mice were treated with PBS, rmIL-18 Fc chimera, and rmIL-18 respectively for four days and were sacrificed for organ collection on day 5 post inoculations and treatment. 

**Fig. 1:**
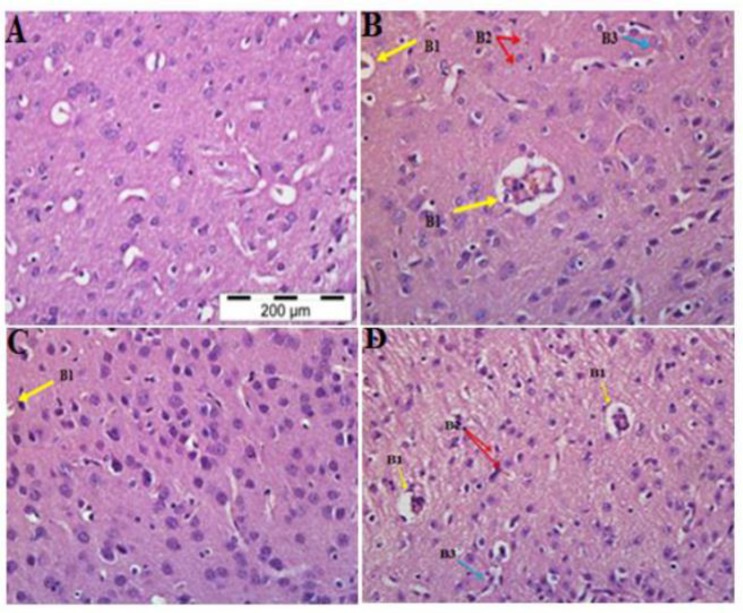
H&E staining of brain Tissue in the PBS-treated control mice (A), PBS-treated (B), rmIL-18Fc Chi mera-treated (C) and rmIL-18-treated (D) malarial mice. In the all malarial mice abnormal features such as Sequestration of PRBCs in the cerebral microvessels (B1, yellow arrow), pigmentation (B2, red arrow) and diffuse inflammatory cells (B3, blue arrow) were observed as compared to the control mice (A). This pathologi cal feature was less significant in the malarial mice treated with rmIL-18Fc Chimera but no signifi cant differences were observed in rmIL-18-treated malarial mice (H&E staining, 400x magnification)

**Brain:** Several histopathological changes were observed in the brain of PBS- treated malarial mice. There was sequestration of PRBC in the cerebral microvasculature and diffuse inflammatory cells. Small and activated lymphocytes were observed in surrounding tissue of the brain. Haemozoin-containing red blood cells in the blood vessels, which were characterized by the brownish small spots, was observed in the blood vessels of cerebral cortex region of the brain in all malarial mice treated with PBS (Fig.1B). Both groups of rmIL-18 and rmIL-18 Fc chimera-treated malarial mice showed pigmentation and sequestration of PRBCs in the microvasculature and sparse inflammatory cell infiltration (Fig. 1C and Fig. 1D), a feature similar to those of PBS-treated malarial mice. Interestingly, the brain tissues of all malarial mice treated with rm-IL-18 Fc chimera did not show severe lesion as compared to malarial mice treated with PBS and rmIL-18.

**Fig. 2:**
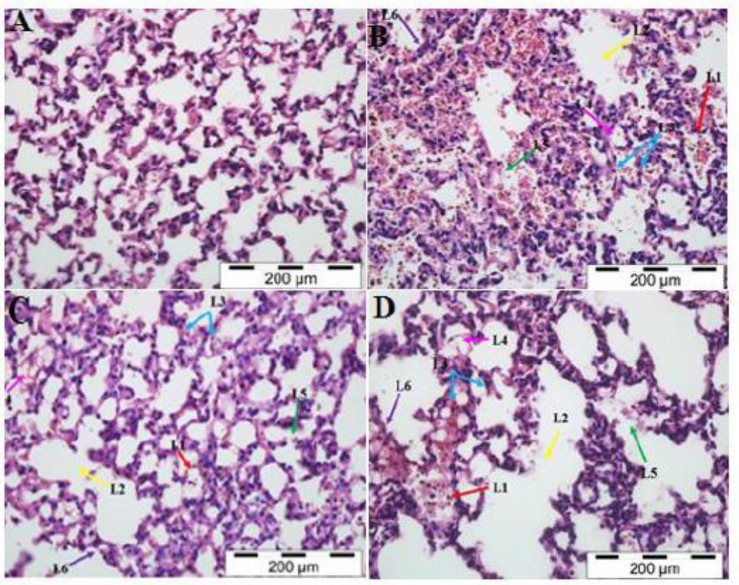
H&E Staining of Lung Tissue in the PBS-treated Control Mice (A), PBS-treated (B), rmIL-18Fc Chimera-treated (C) and rmIL-18-treated (D) Malaria Mice. Sequestration of PRBCs (L1, red ar row), architecture loss (L2, yellow arrow), pigmentation or accumulation of haemozoins in the interstit ium and intralveolar spaces (L3, blue arrow) of lungs tissues, pulmonary edema (L4, pink arrow), Hyaline membrane formation due to alveolar haemor rhage (L5, green arrow), and thicked alveolar septa (L6, purple arrow) were observed in malarial mice treated with PBS, rmIL-18Fc Chimera and rmIL-18 as compared to control mice (A). Malarial mice treated with rmIL-18Fc Chimera showed im proved morphological and histopathological condi tions changes (C). (H&E staining, 400x)

**Lung:** Lung tissues of normal uninfected mice show clear alveolar walls as shown in Fig. 2A and the lung tissues of all malaria-infected mice treated with PBS, collected on day 5 post inoculation showed features such as sequestration of PRBCs and pigmentation in the interstitium and intralveolar spaces of lung tissue, architecture degeneration, pulmonary edema and hyaline membrane formation in the alveolar walls due to alveolar hemorrhages and thickened alveolar septa (Fig. 2B).

In Fig. 2C, no hyaline membrane formation was observed in all malarial mice treated with rmIL-18 Fc chimera and the alveolar septa appearance was also normal as compared to PBS-treated malarial mice. Moreover, there were significantly less sequestration of PRBCs and haemozoins accumulation in the interstitium and intra-alveolar spaces of lung tissue, pigmentation and pulmonary edema as well as improvement on architecture of lung tissue in the malarial mice treated with rmIL-18 Fc chimera. Nevertheless, severe architecture loss and thickened alveolar septa were observed in the group of animals treated with the rmIL-18. Both group of PBS and rmIL-18-treated malarial mice showed almost similar histopathological features. 

**Fig. 3:**
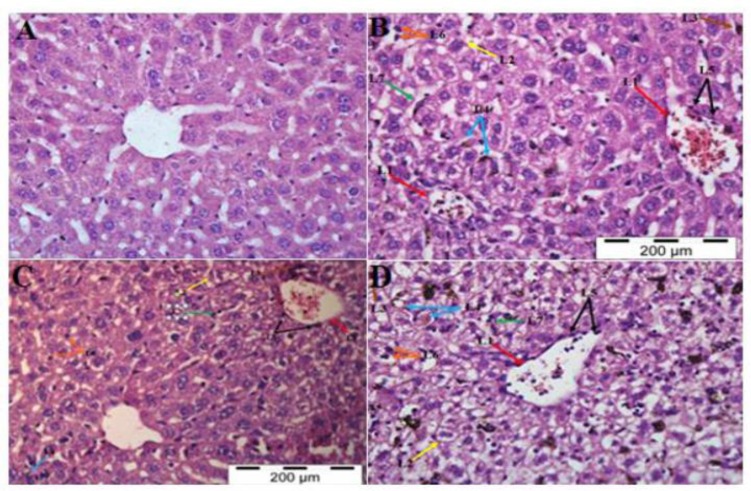
H&E Staining of Liver Tissue in the PBS-treated Control Mice (A), PBS-treated (B), rmIL-18Fc Chimera-treated (C) and rmIL-18-treated (D) Malaria Mice. Sequestration of microvascular with PRBCs (L1, red arrow), architecture loos (L2, yellow arrow), necrosis of centrilobular regions (L3, brown arrow), pigmentation (L4, blue arrow), lymphocytic infiltration identified around centrilobular veins (L5, black arrow), hyperplasia and hypertrophy of kupffer cells (L6, orang arrow) with vacuolar degeneration and atrophy of hepatocytes leading to hepatomegaly, and hyperemia accompanied with the dilatation of the vascular channels (sinusoids) (L7, green arrow) were ob served in the liver of malaria-infected mice treated with PBS, rmIl-18Fc chimera and rmIL-18, as compared to con trol mice (A). Treatment with rmIL-18Fc Chimera significantly improved the histopathological conditions (C). (H&E staining, 400x)

**Liver:** Liver of malarial mice treated with PBS showed severe malaria histopathological features such as sequestration of PRBCs in the blood vessels, necrosis and degeneration of centrilobular regions, and accumulation of malaria pigments in sinusoids and centrilobular veins. Lymphocytic infiltrations were also observed around the centrilobular veins causing inflammation, hypertrophy and hyperplasia of Kupffer cells with vascular degeneration and atrophy of hepatocytes leading to hepatomegaly and architecture loss as well as hyperemia (Fig. 3B). Liver tissues from rmIL-18 Fc chimera-treated malarial mice showed significantly improved morphological features as compared to the PBS treated malarial mice. There were less architecture loss, less sequestration of the blood vessels with PRBCs, less pigmentation and hypertrophy and hyperplasia, mild necrosis and degeneration of centrilobular (Fig. 3C). Liver sections obtained from mice treated with rmIL-18 however revealed histopathological features that were similar to those of PBS-treated malarial mice.

**Fig. 4:**
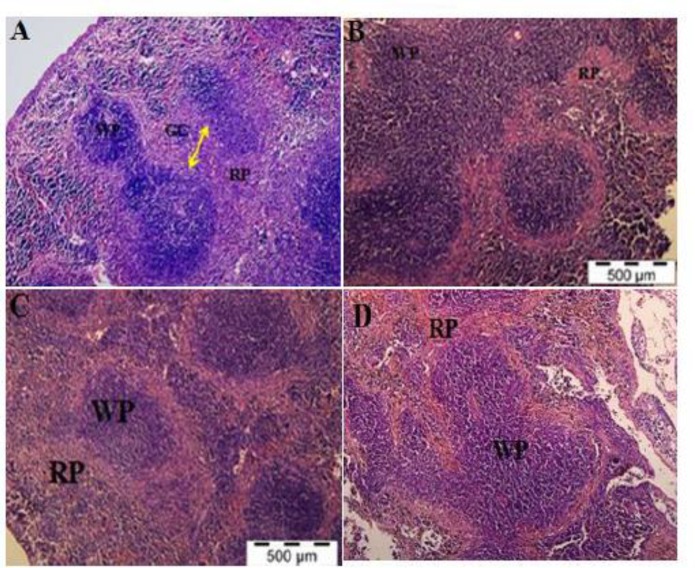
H&E Staining of Spleen Tissue in the PBS-treated Control Mice (A), PBS-treated (B), rmIL-18Fc Chi mera-treated (C) and rmIL-18-treated (D) Malaria Mice. Spleen of infected mice treated with PBS and rmIL-18 showed enlargement of red and white pulp elements accompanied by loss of typi cal structure of germinal center leading to splenomeg aly (B, C and D). Malarial mice treated with rmIL-18Fc chimera showed improved morpho logical and histopathological changes (C) (H&E staining, 100x)

**Spleen:** In the spleen of normal uninfected control mice, clear distinction between red and white pulp, resting follicles and marginal zone was evident (Fig. 4A). In contrast, malaria-infected mice lost their typical structure of germinal center accompanied with enlargement of red and white pulps during the infection period (Fig. 4B). 

**Fig. 5:**
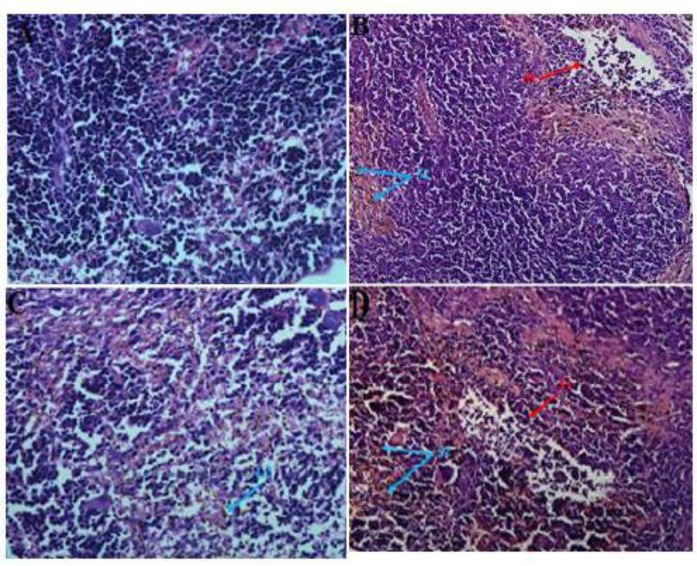
H&E Staining of Spleen Tissue in the PBS-treated Control Mice (A), PBS-treated (B), rmIL-18Fc Chi mera-treated (C) and rmIL-18-treated (D) Malaria Mice. Sequestration of haemozoins and PRBCs in the microvas cular (S1, red arrow) and accumulation of malarial pigments were deposited abnormally in the red pulp histiocytes, sinusoidal lining areas (S2, blue arrow) as observed in (B), (C) and (D). No severe pathologi cal changesidentified in malaria treatment mice with rmIL-18Fc Chi mera(C) (H&E staining, 400x)

In addition, other abnormal features were also observed in the splenic tissues of malarial mice, which include sequestration of PRBCs in the microvascular and an abundance of malarial pigments deposition in the pulp histiocytes and sinusoidal lining (Fig. 4B). All malarial mice treated with rmIL-18 Fc chimera showed improved morphological and histopathological features than those exhibited by the PBS-treated malarial mice upon microscopic examination of their splenic tissues on day 5 post inoculation. Spleen sections of mice treated with rmIL-18 Fc chimera showed less architecture loss as well as mild congestion of PRBCs in the microvasculature. However, similar pigment deposition in the red pulp histiocytes, sinusoidal lining areas were observed in both malarial mice group treated with rmIL-18 Fc chimera and PBS (Fig. 4C and Fig. 5C). 

Nevertheless, inspected spleen sections obtained from mice treated with rmIL-18 revealed severe histological lesions compared to malarial mice treated with PBS. 

**Fig. 6:**
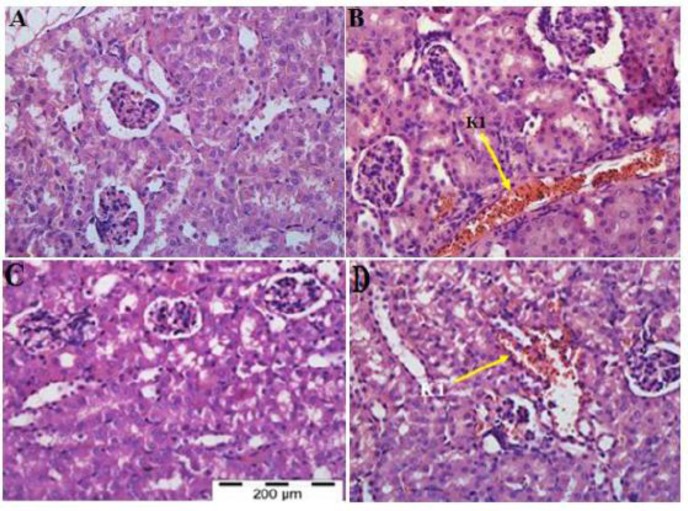
H&E Staining of Kidney Tissues Cortex in the PBS-treated Control Mice (A), PBS-treated (B), rmIL-18Fc Chi mera-treated (C) and rmIL-18-treated (D) Malaria Mice. Severe sequestration of PRBCs and haemozoins in the blood vessels (K1, yellow arrow) by PRBCs was presented in cortex of all malaria-infected (B), (C) and (D). No severe histopathologi cal changes identified in malaria treatment mice with rmIL-18Fc Chimera (C) (H&E staining, 400x)

**Fig. 7:**
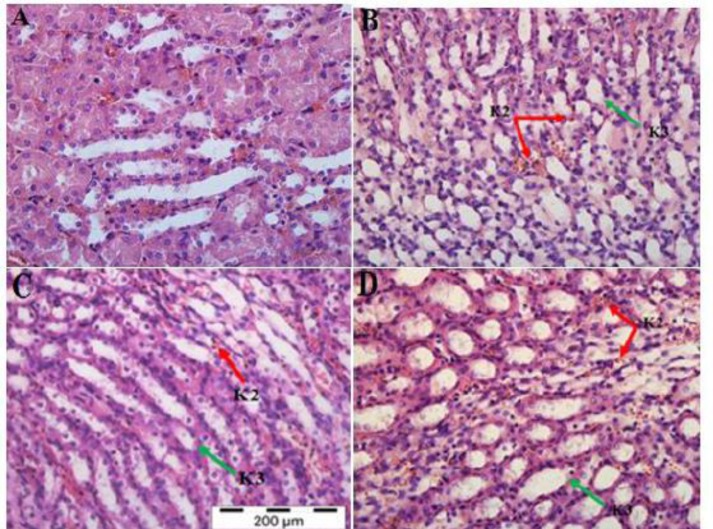
H&E Staining of Kidney Tissues Medulla in the PBS-treated Control Mice (A), PBS-treated (B), rmIL-18Fc Chi mera-treated (C) and rmIL-18-treated (D) Malaria Mice. Kidney’s medulla from infected mice was presented with wide spread of haemozoin (malaria pigment) in the interstitum (K2, red artow). Vaculation of the tubules (K3, green arrow) were exhibited as well in (B), (C) and (D).Mild pathological changes identified in malaria treatment mice with rmIL-18Fc Chimera (C) (H&E staining, 400x)

**Kidney:** The kidney tissues of the normal uninfected control mice showed normal kidney cells, with clear, clean glomeruli, and RBC when compared with the malarial mice treated with PBS (Fig. 6A and Fig. 7A). However, the cortex and the medulla part of the kidney of all malarial mice treated with PBS, presented with severe sequestration of PRBCs in the blood vessels, widespread of pigmentation in the interstitial tissue as well as vacuolation of the tubules (Fig. 6B and Fig. 7B). Treatment of malarial mice with rmIL-18/Fc chimera significantly reduced the severe histopathological features occurring during malaria infection (Fig. 6C and 7C). Sections of kidney taken from malarial mice treated with rmIL-18 Fc chimera showed significant reduction in the sequestration, pigmentation and tubular vacuolation compared to malarial mice treated with PBS. However, malarial mice treated with rmIL-18 exhibited similar histological features of PBS-treated group (Fig. 6D and 7D). 

## Discussion

Malaria parasites accumulated not only in the peripheral circulation but also in the microvasculature of internal organs such as brain (16), lungs (17), liver (18), spleen (19) and kidneys (20). Previous studies revealed that sequestration of PRBCs could lead to microvascular damage in the brain tissue of *P. berghei*-infected mice (21, 22). One of the most common pathological changes observed in the brain tissues of *P. berghei*-infected mice involved cerebral lesions that can lead to neurological impairment (23). The cerebral lesions in animals were usually presented with sequestration of PRBC or congestion, and haemozoins that block the microcapillaries. During human cerebral malaria, these marked changes were typically observed. The intravascular congestion with inflammatory infiltrates in the microcapillaries suggested that there might be an immunological response occurs at the surface of the infected red blood cells due to parasite invasion. The existence of hemorrhages may demonstrate that the congested blood vessels have become unusually delicate (24). In this study, treatment of malarial mice with rmIL-18 did not show any difference as compared with malaria-infected mice since there was severe congestion of malarial pigments observed following treatment with this drug. However, the tissues of all the treated mice with rm-IL-18 Fc chimera significantly showed moderate lesion as compared with malarial mice treated with PBS. According to previous studies, the sequestration of the PRBCs is associated with hyperactivity of Th1 cell and release of pro-inflammatory cytokines such as IFN-, TNF-α, IL-1α and IL-6 (25-27). The release of these cytokines contributed towards the up-regulation of NO and ICAM-1 production, resulting in microvascular blockage and neurotransmission interruption (25, 28, 29). This suggests that the sequestration of PRBCs in the brain of malaria-infected mice treated with PBS and rmIL-18 is contributed by the up-regulation of the pro-inflammatory cytokines during malaria infection. Thus, the results suggest that IL-18 is involved in the development of experimental CM and maybe one of the key mediators of the disease severity.

Several previous studies demonstrated the pulmonary complications in lung tissues following malaria infection (30-32). Inflammation of endothelial cell and infiltration of inflammatory cells such as monocytes together with PRBCs adherence within the capillaries, leading to septal or interstitial oedema in the lungs were detected during *falciparum *malaria (33, 34). Furthermore, the histological analysis of the lungs in rodent malaria model demonstrated endothelial adhesion of pigment-containing monocytes and neutrophils accompanied by septal pneumonitis, resulting in the increased pulmonary vascular permeability (30, 31, 35). The hyaline membrane formation in the alveoli of lungs tissues of malarial mice, recommended that there was leakage of proteinaceous fluid (36).

Our results were in accordance with prior study that verified mononuclear cells and pigment-laden macrophages were always seen amidst the PRBC in the microvessels of alveolar septa, suggesting that injury in pulmonary capillaries were caused by the release of toxic mediators from adherent mononuclear cells (37). The damage of alveolar-capillary membrane function leads to reduced gas transfer and eventually worsens the disease condition (38). These features are consistent with a previous observation, in which the lung tissues of *P. berghei-*infected mice were presented with increased number of inflammatory cells in the alveolar septa, which may indicate acute lung injury in malarial mice (39). The lung damage in *P. berghei-*infected mice indicated several similar features of severe human malaria infection, whereby both models presented accumulation of PRBCs in the alveolar-capillary membrane barrier and pulmonary edema (39).

Moreover, there were abundant of haemozoin and PRBC deposited in the interstitium and in the intralveolar spaces of all malarial mice treated with PBS and recombinant IL-18 and minimal PRBCs congestion were observed upon treatment with rmIL-18 Fc chimera. The congestion of intravascular spaces with PRBCs and haemozoins might correlate with the parasitaemia levels of malarial mice. As the parasitaemia level amplified, deposition of PRBCs and malaria pigments in the lung tissue intensify. This result is in accordance with earlier work, which proposed an effect of the peripheral parasitaemia in causing lung damage during rodent model of malaria infection (39). Additionally, lung injury observed in the mice may be contributed by the release of inflammatory cytokines. The release of IFN-, TNF-α, IL-1α and IL-6 in the plasma of malarial mice was associated with the inflammation of the lung tissues, leading to acute lung damage (39). Taken together, the results showed that lung damage in *P. berghei*-infected mice is the consequences of both PRBCs sequestration in the lungs tissues and excessive inflammatory response to clear the malarial parasites, suggesting that IL-18 possible involvement in the lung pathology during malaria infection.

Hyperplasia and hypertrophy of Kupffer cells seen in liver cells were due to phagocytotic process to eliminate the parasites and the enlargement as well as increased in number of Kupffer cells in the liver were due to the active phagocytic process by the host immune responses to eliminate the parasites. These abnormalities contribute to hepatomegaly and architecture loss, a common feature of liver damage in the malaria-infected mice (40, 41). The presence of black pigments in the hepatocytes resulted from congestion of haemozoins (40). Hepatic dysfunction during *Plasmodium berghei* ANKA infection may elucidate the malarial pigments accumulation in the liver sections (42). During malaria infection, the most significant observation was accumulation of PRBCs in the sinusoids of malaria-infected mice, resulting in congestion of the microvessels in the liver of all malaria mice. This feature expresses the black pigmentation resulted from the congestion of haemozoins in the sinusoids (40). The malaria pigments accumulation in the liver tissues also clarifies the hepatic dysfunction during *P. berghei *ANKA infection. Additionally, the parasites sequestration in the liver microvasculature correlates with the increase in liver enzymes, aspartate transaminase (AST), indicating parasites role in mediating hepatic dysfunction (42, 43). 

Hyperemia with dilatation of vascular channels in the liver tissues may be correlated with the decreased in blood osmotic pressure and finally decreased the drainage of tissue fluids causing oedema (44). Previous study discovered that haemozoin has been shown to stimulate the release of cytokines and parasite cytoadherence during malaria infection (45). The degree of the lesion in the liver is correlated with the amount of haemozoins in the tissues (46). Microscopically, lymphocytic infiltration around centribular vein was observed in the liver tissues of all malarial mice. It has been shown in earlier studies that migration of the haemozoins from the parenchymal to the portal areas leads to lymphocytic infiltration around the centribular vein of the liver (41, 47, 48). The high concentration of these cytokines in the plasma of treated-malarial mice may reflect the pathological changes observed in the liver sections. Moreover, the histopathological analysis by previous scientists showed that parasite burden is related to the liver damage in malaria-infected hosts (42, 49)

The overall enlargement of red and white pulps in the spleen sections were associated with the loss of germinal center typical structure, resulted from hyperactivity of the macrophages to fight against the malaria parasites (50). In accordance with the previous microscopic results in the spleen, the widespread of malarial pigments in the microvascular, red pulp area and sinusoidal lining areas was found to be consistent with the elevated parasitaemia level in the infected host. Moreover, a higher parasitaemia level is associated with higher amounts of free haemoglobin, capable of releasing more malaria-infected haem (51, 52). Besides that, higher pigmentation could further impair the macrophage function (53) and trigger the host immune system to release more TNF-α (54). Since the amount of haemozoins spread was inconsistent with parasitaemia level, it is suggested that the high parasitaemia level measured in rmIL-18-treated malarial mice was resulted from an acute splenic response towards malaria infection whereas the reduced parasitaemia level in rmIL-18 Fc chimera-treated malarial mice and the enlargement of red and white pulps indicate an effective splenic filtration in the mice during the infection. In addition, the release of pro-inflammatory cytokines such as IFN- by the dendritic cells (DCs) may have caused splenic tissue abnormalities as observed in the treated mice, proposing that the activated DCs in the splenic tissues promotes filtration of PRBCs and trigger the immune responses (55). In this study, there were significant differences in the histopathological analysis of spleen sections between malarial mice treated with rmIL-18 Fc chimera and PBS which suggested that inhibition of IL-18 improved the pathological conditions of the spleen by down-regulating the pro-inflammatory cytokines.

Renal dysfunction is commonly related with cerebral involvement, high parasitaemia, jaundice and haemoglobinuria (55, 56). The widespread of malarial pigments, PRBC and monocytes, observed in the blood vessels and interstitial tissues, has been associated with reduced microvascular blood flow which eventually causing renal impairment and ischaemic nephropathy (57). The presence of tubular vacuolation may propose that the infected mice suffered from black water fever, an acute hemolytic condition associated with fever, jaundice, anemia and hemoglobinuria (57). The kidney pathologies observed in the rmIL-18 treatment groups may be associated with the cytokines release induced by increased IL-18 production. Previous study indicated that the hyperproduction of pro-inflammatory cytokines and the dysregulation of anti-inflammatory cytokines contributed towards the kidney pathology during malaria infection. This was supported by the several-pigmented macrophages, presenting positive staining both for pro- and anti-inflammatory cytokines in the tubulointerstitium (58). This preliminary histology results showed that rmIL-18 Fc chimera might have a potential role in reducing further tissue damage in the affected organs during malaria infection. It may also serve as an important additional therapeutic approach in endemic areas of malaria that has shown resistance towards the conventional antimalarial therapy.

## Conclusion

All tissues from the organs of malarial mice treated with rmIL-18 Fc chimera were presented with significant improvement on their histopathological conditions as compared to the malarial mice treated with PBS. Nonetheless, following treatments with rmIL-18, the treated malarial mice displayed similar histopathological changes with PBS-treated malarial mice, except for minor differences in the lung and spleen. It can therefore be concluded that IL-18 is involved in mediating the severe pathological conditions associated with malaria infection and the host may benefit from its antagonism. 
